# Effects of socioeconomic status and race on survival and treatment in metastatic breast cancer

**DOI:** 10.1038/s41523-023-00595-2

**Published:** 2023-11-01

**Authors:** Susrutha Puthanmadhom Narayanan, Dianxu Ren, Steffi Oesterreich, Adrian V. Lee, Margaret Q. Rosenzweig, Adam M. Brufsky

**Affiliations:** 1 UPMC Hillman Cancer Center, Pittsburgh, PA USA; 2 Women’s Cancer Research Center (WCRC), UPMC, Pittsburgh, PA USA; 3 University of Pittsburgh, Pittsburgh, PA USA; 4 Magee-Womens Research Institute, Pittsburgh, PA USA

**Keywords:** Breast cancer, Breast cancer

## Abstract

Race and socioeconomic factors affect outcomes in breast cancer. We aimed to assess the effect of race and neighborhood socioeconomic status (SES) on overall survival and treatment patterns in patients with metastatic breast cancer (MBC). This is a retrospective cohort study involving patients (*N* = 1246) with distant breast cancer metastases diagnosed at UPMC Magee Women’s Breast Cancer Clinic from 2000–2017. Overall survival and treatment patterns were compared between races (Blacks and whites) and SES groups (defined using Area Deprivation Index). Low SES, but not tumor characteristics, was associated with Black race (*P* < 0.001) in the study population. Low SES (Median [Interquartile Range, IQR] survival 2.3[2.2–2.5] years vs high SES 2.7[2.5–3.1] years, *P* = 0.01) and Black race (Median [IQR] survival 1.8[1.3–2.3] years, vs white 2.5[2.3–2.7] years *P* = 0.008) separately were associated with worse overall survival in patients with MBC. In the Cox Proportional Hazard model with SES, race, age, subtype, number of metastases, visceral metastasis, and year of diagnosis as covariates, low SES (Hazard ratio 1.19[1.04–1.35], *P* = 0.01), but not Black race (Hazard ratio 1.19[0.96–1.49], *P* = 0.12), independently predicted overall survival in MBC. Moreover, patients from low SES neighborhoods and Black race received fewer lines of chemotherapy than high SES and whites. In conclusion, low neighborhood SES is associated with worse outcomes in patients with MBC. Poor outcomes in Black patients with MBC, at least in part is driven by socioeconomic factors. Future studies should delineate the interplay between neighborhood SES, race, and their effects on tumor biology in MBC.

## Introduction

Racial disparities exist in the outcomes of breast cancer in the United States. The age-adjusted breast-cancer mortality among Black women (27.7 deaths per 100,000) is 40% higher than among non-Hispanic white women (20.0 per 100,000) from 2014 through 2018^1^. Several factors influence the outcomes in Black patients with breast cancer. These include biologic factors such as a higher proportion of hormone negative and high-grade disease^2^, as well as socioeconomic and cultural factors such as inadequate health insurance, poor access to health care, a general mistrust of the health care system and social injustice related to racial profiling and discrimination^3^. Outcomes in metastatic breast cancer (MBC) are also affected by race and socioeconomic status (SES). A study based on the Surveillance, Epidemiology, and End Results (SEER) database showed that low neighborhood SES is associated with higher mortality among patients with de novo MBC^4^. The mortality risk was also substantially higher in non-Hispanic Black than non-Hispanic white women with de novo MBC in the SEER database^5^. However, this increased mortality risk was mitigated after adjusting for socioeconomic, tumor and metastatic characteristics suggesting a complex interplay of social and biologic factors in mediating racial disparities in breast cancer.

The Area Deprivation Index (ADI) is a powerful tool used to estimate the area-level SES^6,7^. It is a comprehensive tool that includes multiple domains such as education, income, household composition, home value, household facilities, poverty, and employment. In the current study, we aimed to assess the effect of race and neighborhood SES on survival of patients with MBC at our center, the UPMC Magee Women’s Breast Cancer Clinic. In contrast to prior studies, we included patients with both de novo and recurrent MBC and used the ADI score to define neighborhood SES. We also examined the utilization of different treatment options in these patients.

## Results

### Description of study population

Demographic and clinical features of MBC are described for the SES categories (low SES, *N* = 832 and high SES, *N* = 414) and racial groups (Black, *N* = 100 and white, *N* = 1111) in Table 1. Patients from low SES neighborhoods were slightly younger than those from high SES neighborhoods (Mean [SD]: 56.1 [12.8] in low SES and 57.6 [13.0] in high SES, *P* = 0.049). There was a significant association between low SES and Black race (10.5% Blacks in low SES and 3.7% in high SES, *P* < 0.001). None of the other parameters including subtype, being de novo versus recurrent, number of metastases was associated with SES. In the comparison by race, the proportion of triple negative disease was higher in Blacks than whites, but this was not statistically significant (27.1% in Blacks and 21.3% in whites, *P* = 0.13).

**Table 1:** Demographics, clinical features and treatments received in MBC patients by socioeconomic status and race.

	Low SES (*N* = 832)	High SES (*N* = 414)	*P*-val	Black (*N* = 100)	White (*N* = 1111)	*P*-val
Age, mean(SD)	56.1 [12.8]	57.6 [13.0]	0.049	56.4 [12.4]	56.6 [13.0]	0.89
Race (Black, %)	10.5	3.7	<0.001	NA	NA	NA
Missing (N)	22	13		NA	NA	
Histology (D, L in %)	80, 10	76, 12	0.5	77, 9.3	79, 10.7	0.39
Missing (N)	55	25		3	73	
Denovo mets, Yes N,%	182, 21.9	93, 22.5	0.87	23, 23%	244, 22%	0.9
Subtype (%)						
ER + /HER2+	12.3	17.6	0.1	9.4	14.3	0.13
ER-/HER2+	8.9	8.1		4.2	9.1	
ER + /HER2-	56.8	53.9		59.4	55.3	
ER-/HER2- (Triple Negative)	22	20.4		27.1	21.3	
Missing (N)	45	21		4	55	
Number of metastases, median [IQR]	3 [2–4]	3 [2–4]	0.84	3 [2–4]	3 [2–4]	0.9
Visceral metastases at diagnosis, Yes %	39	41	0.7	40	40	>0.99
Missing (N)	0	1		0	0	
Utilization of different treatment options in MBC						
Chemotherapy in all MBC (Yes %)	83	83	0.99	77	83	0.12
Missing (N)	5	2		1	2	
Chemotherapy in Triple negative MBC (Yes %)	93	97.5	0.25	92.3	94	0.96
Missing (N)	0	1		0	0	
Lines of chemotherapy in MBC, Median[IQR]	2 [1,4]	3 [1–5]	0.19	2 [1,3]	3 [1,5]	0.03
Missing (N)	5	2		1	2	
Lines of chemotherapy in Triple negative MBC, Median[IQR]	3 [2–5]	3 [2–5]	.37	3 [2.4]	3 [2,5]	0.9
Missing (N)	0	1		0	0	
HER2 targeted therapy in HER2 + patients (Yes %)	90	87	0.34	85	89	>0.99
Missing (N)	2	0		1	1	
Hormone therapy in ER + patients (Yes %)	91	91	0.9	94	91	0.29
Missing (N)	3	0		1	1	
Tamoxifen use in first line hormone therapy in ER + patients (Yes %)	18	13	0.07	18	16.7	0.97
Missing (N)	70	38		7	97	

Continuous variables are compared between groups using Wilcoxon test, categorical variables are compared between groups using chi-square test, *P* < 0.05 is considered significant.

*SES* Socioeconomic status, *SD* Standard Deviation, *IQR* Interquartile Range.

### Survival by SES groups and race

**Fig. 1:**
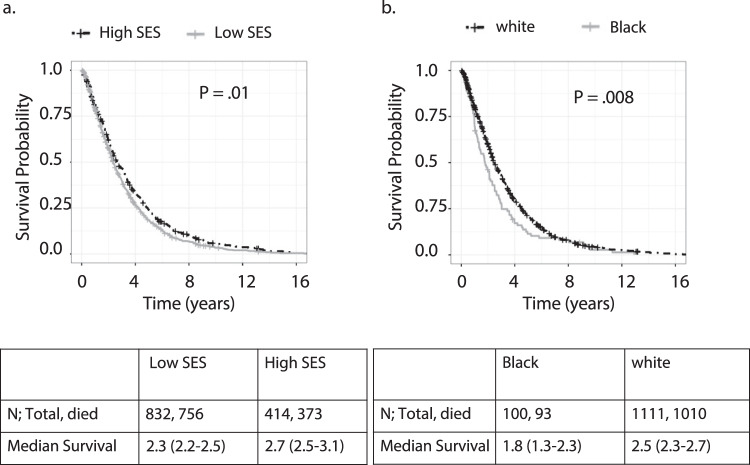
Kaplan–Meier survival plots for patients with metastatic breast cancer. **a** Low vs high SES groups and (**b**) Black vs whites. Logrank test *P*-values reported SES Socioeconomic status.

Univariate Kaplan–Meier analysis revealed that patients living in low SES neighborhoods and patients from Black race had worse overall survival than patients from high SES neighborhoods and white race respectively (Median [IQR] survival for low SES 2.3 [2.2–2.5] years, high SES 2.7 [2.5–3.1] years, *P* = 0.01; Black 1.8 [1.3–2.3] years, white 2.5 [2.3–2.7] years, *P* = 0.008, Fig. 1). In the multivariate Cox Proportional Hazard model using SES group, race, age, subtype of breast cancer, number of metastases, presence of visceral metastasis at diagnosis, and year of MBC diagnosis (before vs after 2010) as covariates, we found that low neighborhood SES was an independent predictor of overall survival (Hazard ratio for low SES 1.19 [1.04–1.35], *P* = 0.01, Table 2). However, race was no longer a significant predictor of survival in this analysis (Hazard ratio for Black race, 1.19 [0.96–1.49], *P* = 0.12).

**Table 2:** Cox Proportional Hazard model for overall survival in metastatic breast cancer.

	Hazard ratio [95% CI]	*P*-value
Race = Black, Ref white	1.19 (0.96–1.49)	0.12
SES = low SES, Ref High SES	1.19 (1.04–1.35)	0.01
Age at MBC diagnosis	1.009 (1.004–1.014)	<0.001
Subtype = ER + /HER2-, Ref ER-/HER2+	0.82 (0.66–1.03)	0.09
Subtype = ER + /HER2 + , Ref ER-/HER2+	0.75 (0.57–0.97)	0.03
Subtype = Triple negative, Ref ER-/HER2+	1.77 (1.39–2.26)	<0.001
Number of metastases	1.01 (0.97–1.06)	0.6
Visceral metastases at diagnosis = Yes, Ref No	1.19 (1.05–1.34)	0.007
Diagnosis of MBC after 2010, Ref before 2010	1.66 (1.45–1.91)	<0.001

*SES* Socioeconomic Status, *CI* Confidence Interval, *MBC* Metastatic Breast Cancer, *Ref* Reference.

### Treatment patterns in MBC by SES group and race

We compared the utilization of chemotherapy, hormone therapy and HER2 targeted therapy between the SES and racial groups (Table 1). The proportion of patients with MBC receiving chemotherapy was not different between the SES and racial groups. However, the number of lines of chemotherapy was significantly different between the racial groups such that Blacks received fewer lines of chemotherapy as compared to whites (Median [IQR] lines of chemotherapy in Blacks 2[1,3] and whites 3[1,5], *P* = 03). We further tested this in a Poisson regression model correcting for SES group, age at MBC diagnosis, year of MBC diagnosis and subtype. Black race and low SES were significant predictors of treatment with fewer lines of chemotherapy in the multiple Poisson regression model (Black race, Incidence Rate Ratio, IRR = 0.81, *P* = 0.002; low SES, IRR = 0.9, *P* = 0.005; Table 3). The proportion of patients with HER2 + MBC receiving HER2 targeted therapy and the proportion of patients with ER + MBC receiving any hormone therapy was not different between the SES or racial groups. Among patients with ER + MBC, treatment with tamoxifen (compared to aromatase inhibitors +/- newer agents) was more commonly reported among patients from low SES (18% in low SES and 13% in high SES, *P* = 0.07), but this was not statistically significant.

**Table 3:** Poisson regression model predicting number of lines of chemotherapy in patients with MBC.

	Incidence Rate Ratio (IRR)	*P*-value
Age at diagnosis	0.98	<0.001
Race = Black, Ref = white	0.81	0.002
SES = low SES, Ref = High SES	0.91	0.005
Diagnosis of MBC after 2010, Ref before 2010	0.99	0.79
Subtype = ER + /HER2-, Ref ER-/HER2+	0.84	0.002
Subtype = ER + /HER2 + , Ref ER-/HER2+	1.02	0.75
Subtype = Triple negative, Ref ER-/HER2+	0.99	0.9

*SES* Socioeconomic Status, *MBC* Metastatic Breast Cancer, *Ref* Reference.

## Discussion

There are three major takeaways from the current study—(1) SES is an independent predictor of overall survival among patients with MBC—low neighborhood SES was associated with worse overall survival even after correcting for potential covariates; (2) Race was no longer a significant predictor of survival in MBC after correcting for SES and tumor characteristics; (3) There are disparities in MBC treatment patterns between SES and racial groups.

The ADI score was used to define neighborhood SES in this study. It is a comprehensive tool encompassing several domains of neighborhood SES. However, it is difficult to ascertain which specific factors within the ADI drove the difference in survival noted in the study. Indeed, uninsured status was associated with early death (within 6 months of diagnosis) in patients with de novo MBC in a prior population-based study^8^. Variability in access to healthcare and practice patterns by geography also contributes to disparities by neighborhood SES^9,10^. Breast cancer related financial burden disproportionately affects low-income patients and racial/ethnic minorities^11^. Yet another mechanism that has gained attention recently is the deleterious biological effects of long-term exposure to poverty, discrimination, and stress. Increased expression of Conserved Transcriptional Response to Adversity genes and higher allostatic load are associated with social isolation, low SES, systemic racism, and discrimination among women with breast cancer and likely influence cancer development and progression^12,13^.

In our study, race was not a significant predictor of survival in MBC after correcting for potential covariates, most notably SES. Akin to our results, a large population-based study using the SEER database also revealed that the higher MBC-related mortality risk seen in non-Hispanic Black women (as compared to non-Hispanic white women) was mitigated in the fully adjusted model^5^. The estimated proportion of excess risk attributed to socioeconomic factors was 66.5%, followed by tumor characteristics at 41.5%, and the metastatic pattern at 14.8%^5^. We found a significant association between race and SES in our database such that 10.5% of patients in low SES neighborhoods were Black while only 3.7% of patients in the high SES neighborhoods were Black (*P* < 0.001). Unlike in prior studies^1^, the percentage of triple negative disease was not significantly higher in Black than white in our database. In the absence of a significant association between Black race and other demographic and tumor characteristics, we believe that the loss of significance of race as a predictor of survival in the multivariate analysis is, at least, in part driven by SES.

Racial and socioeconomic disparities also affect treatment patterns in breast cancer^14–16^. Prior studies reported lower odds of receiving adjuvant chemotherapy and clinically significant delays in initiating adjuvant chemotherapy among Black patients as compared to white patients with early-stage breast cancer^17,18^, but this was not studied in the metastatic setting. In the current study, Black race and low SES independently predicted treatment with fewer lines of chemotherapy for MBC. Financial toxicity, treatment delays, higher symptom distress, and its negative impact on the ability to receive on-time and full-dose chemotherapy^19^ could all be contributing to racial and socioeconomic disparity in breast cancer treatment.

The main strength of the study is that it included patients with both de novo and recurrent MBC unlike prior studies on disparities which were restricted to de novo MBC. Recurrent MBC constituted a major fraction of our study population (~78%), similar to a prospective study from another academic cancer center^20^, enabling us to assess disparities in this group of patients. The sample size was large for a single center study, and a powerful and comprehensive tool—the ADI score, was used to summarize neighborhood SES. Since this is a single center study, variability in practice patterns is less likely to affect the results.

A major limitation of this study is the low proportion of Black patients (8%) as compared to prior population-based studies (SEER MBC database has 17% Blacks)^5^. This could have driven the lack of a significant difference in tumor characteristics between Blacks and whites, particularly the proportion of triple negative MBC. ER and/or HER2 status was not available in 5% (66 out of 1246) of patients, hence reducing the sample size available for analysis involving tumor subtypes. Among 268 patients with HER2 + disease, 27 (10%) did not receive HER2 directed therapy. This is a high percentage of deviation from guideline-based treatment since Trastuzumab was approved in 1998. Of the 27 patients, 9 had HER2- disease in the metastatic tissue, 3 patients did not receive treatment because they had debilitating disease and died within ~1 month of diagnosis of MBC. Another 4 patients did not receive treatment due to patient preference/comorbidities. No reason was available on review of the corresponding patient charts for the rest of the patients. ADI is a comprehensive tool that encompasses several domains of SES, but the database does not have details of individual SES domains such as insurance status/type, income, housing situation or education. This makes it challenging to know which specific domains contributed to the disparity in outcomes, and hence which domains to focus on to improve outcomes in the low SES and Black populations. ADI scores were validated for census block group data, hence, ZIP-code may not be the ideal geographic unit to define a “neighborhood”. Comorbidities are a major potential confounder in survival analysis since disparities in healthcare affect outcomes of several non-cancer diseases as well. However, we could not include comorbidities as a covariate in the survival analysis due to lack of such information in the database. Type of cancer treatment was not included in the survival analysis because we believe that differences in treatment pattern is one of the mechanisms by which SES and race affect outcomes in MBC. In the survival analysis, a hazard ratio >1 (1.7[1.4–1.9]) was noted for MBC diagnosis after 2010 as compared to before 2010. This is likely due to censoring in patients with more recent diagnosis. This is a clinic-based study with a catchment area restricted to Pittsburgh and nearby counties in western Pennsylvania, hence the results are not generalizable to the whole country.

In conclusion, the current study demonstrated that patients from low SES neighborhoods had worse survival even after correcting for race and tumor characteristics. However, race was not a significant predictor of overall survival in the multivariate survival model. In the absence of an association between tumor characteristics and race in our database, we suspect that SES played a major role in mediating poor outcomes in the Black patients. There were differences in treatment patterns between the SES and racial groups. Taken together, these results call for an organized effort to ensure equal access and delivery of care to patients with MBC at our center as well as in the country. Future studies should delineate the roles of specific aspects of neighborhood SES and tumor biology in mediating poor outcomes in Black patients. Deleterious effects of stress, poverty, and discrimination on tumor biology in breast cancer also needs to be further investigated.

## Methods

### Study population

Data for the current study was obtained from the Metastatic Breast Cancer Database which is a prospectively curated database of patients diagnosed with distant breast cancer metastases at UPMC Magee-Women’s Hospital between January 1, 1999, and December 31, 2021. The study was approved by the UPMC Institutional Review Board (STUDY20030072). The need for informed consent was waived by the review board due to a high proportion of participants being deceased. Each patient entry includes 6 demographic, 30 early-stage tumor and treatment, 18 baseline MBC characteristics, and 10 clinical and treatment characteristics for each MBC treatment. Of 1,428 patients who were diagnosed with MBC at our center between 2000–2017, 1,246 patients with a mean [SD] age of 58.8[12.9] years were included in the study based on availability of ZIP-code and survival data. The national ADI scores for these patients were obtained from the Neighborhood Atlas using ZIP-codes that were mapped to census tracts using ZIP-code crosswalk (https://www.neighborhoodatlas.medicine.wisc.edu). ADI is calculated based on the American Community Survey data obtained from 2014–2018. It is reported as the percentile of deprivation such that a higher score suggests higher deprivation and hence, lower SES. The study population had a median [interquartile range, IQR] national ADI of 66[43–80], and median [IQR] follow up of 2.3[1.1–4.1] years (3.3[1.5–5.6] years for ER + /HER2+ disease and 1.3[0.6– 2.4] years for Triple negative disease).

### Statistical analysis

The study population was divided into 2 groups based on the ADI score—the high SES group comprising of patients from neighborhoods in the lower tertile ADI neighborhoods (ie, ADI $\le$ 51, the 33rd percentile ADI score for the study population) and the low SES group with patients in the upper two tertiles. ADI neighborhoods (ADI > 51). Baseline demographic and clinical features at diagnosis of MBC were compared between the two SES groups, continuous variables were compared using Wilcoxon test and categorical variables were compared using chi-squared test. Primary outcome was overall survival, defined as the duration from diagnosis of MBC to death from any cause. Secondary endpoints evaluated the utilization of treatment options in MBC, such as the proportion of patients receiving chemotherapy, hormonal therapy, or HER2 targeted therapy as well as lines of chemotherapy and type of hormonal therapy received. Effect of race and SES on overall survival in MBC was assessed using the univariate Kaplan–Meier analysis as well as Cox Proportional Hazard model where additional covariates were included—age at MBC diagnosis, subtype based on ER and HER2 status, number of metastases (refers to total number of metastases throughout the course of disease), year of MBC diagnosis (before vs after 2010) and presence of visceral metastasis at MBC diagnosis (visceral metastasis was defined as site of metastasis other than bone, lymph node, chest wall or skin). Multivariate analysis with Poisson regression model was used to assess the effect of different factors on treatment utilization in patients with MBC. All statistical analyses were performed using R statistical software (version 4.0.2).

### Reporting summary

Further information on research design is available in the Nature Research Reporting Summary linked to this article.

### Supplementary information

Reporting Summary

## Data Availability

The de-identified dataset used, and/or analyzed during the current study will be available from the corresponding author on reasonable request through a data-sharing agreement.

## Code availability

The Area Deprivation Index (ADI) is a publicly available research tool. Neighborhood Atlas—Home (wisc.edu). No custom codes or mathematical algorithms other than standard statistical tests were used in the study.
